# Roles of IFN-γ in tumor progression and regression: a review

**DOI:** 10.1186/s40364-020-00228-x

**Published:** 2020-09-29

**Authors:** Dragica Jorgovanovic, Mengjia Song, Liping Wang, Yi Zhang

**Affiliations:** 1 Biotherapy Center, The First Affiliated Hospital of Zhengzhou University, No.1 Jianshe Road, Zhengzhou, 450052 Henan China; 2 State Key Laboratory of Esophageal Cancer Prevention & Treatment, Zhengzhou University, Zhengzhou, 450052 China; 3 Department of Biotherapy, Sun Yat-sen University Cancer Center, Collaborative Innovation Center for Cancer Medicine, State Key Laboratory of Oncology in South China, Guangzhou, 510060 China; 4 Cancer Center, The First Affiliated Hospital of Zhengzhou University, No.1 Jianshe Road, Zhengzhou, 450052 Henan China; 5 Henan Key Laboratory for Tumor Immunology and Biotherapy, Zhengzhou, 450052 China

**Keywords:** IFN-γ, Cancer, Tumor microenvironment, Immunoregulation, Immunotherapy, Tumor regression, Tumor progression

## Abstract

**Background:**

Interferon-γ (IFN-γ) plays a key role in activation of cellular immunity and subsequently, stimulation of antitumor immune-response. Based on its cytostatic, pro-apoptotic and antiproliferative functions, IFN-γ is considered potentially useful for adjuvant immunotherapy for different types of cancer. Moreover, it IFN-γ may inhibit angiogenesis in tumor tissue, induce regulatory T-cell apoptosis, and/or stimulate the activity of M1 proinflammatory macrophages to overcome tumor progression. However, the current understanding of the roles of IFN-γ in the tumor microenvironment (TME) may be misleading in terms of its clinical application.

**Main body:**

Some researchers believe it has anti-tumorigenic properties, while others suggest that it contributes to tumor growth and progression. In our recent work, we have shown that concentration of IFN-γ in the TME determines its function. Further, it was reported that tumors treated with low-dose IFN-γ acquired metastatic properties while those infused with high dose led to tumor regression. Pro-tumorigenic role may be described through IFN-γ signaling insensitivity, downregulation of major histocompatibility complexes, upregulation of indoleamine 2,3-dioxygenase, and checkpoint inhibitors such as programmed cell death ligand 1.

**Conclusion:**

Significant research efforts are required to decipher IFN-γ-dependent pro- and anti-tumorigenic effects. This review discusses the current knowledge concerning the roles of IFN-γ in the TME as a part of the complex immune response to cancer and highlights the importance of identifying IFN-γ responsive patients to improve their sensitivity to immuno-therapies.

## Introduction

Interferon-γ (IFN-γ) is the sole member of the type II interferon family discovered almost 60 years ago. E. Frederick Wheelock was the first to describe IFN-γ as a phytohemagglutinin-induced virus inhibitor produced by white blood cells after they have been stimulated [1]. IFN-γ is a protein encoded by the IFNG gene, composed of two polypeptide chains associated in an antiparallel fashion [2]. In human blood, IFN-γ is present in three fractions with different molecular mass. One fraction represents the active free form of IFN-γ, while the other two are considered mature IFN-γ molecules. The fully synthetized protein is glycosylated at amino termini where the level of glycosylation determines the final weight of the defined fractions [3, 4]. Notably, it has been reported that glycosylation itself does not affect the activity of interferon, but rather prevents its degradation by proteinases. Therefore, this chemical modification increases interferons half-life in the bloodstream and prolongs IFN-γ-mediated effects [5].

From a biological point of view, IFN-γ is a pleiotropic cytokine with antiviral, antitumor, and immunomodulatory functions. Hence, it plays an important role in coordinating both innate and adaptive immune response [6]. In an inflammatory environment, IFN-γ triggers the activation of the immune response and stimulates the elimination of pathogens; it also prevents over-activation of the immune system and tissue damage. This balance is maintained by complex mechanisms which are not yet fully understood [7, 8]. In the tumor microenvironment (TME), IFN-γ consistently orchestrates both pro-tumorigenic and antitumor immunity. IFN-γ acts as a cytotoxic cytokine together with granzyme B and perforin to initiate apoptosis in tumor cells [9, 10], but also enables the synthesis of immune checkpoint inhibitory molecules and indoleamine-2,3-dioxygenase (IDO), thus stimulating other immune-suppressive mechanisms [11–13]. Intriguingly, the contradictory biological and pathological effects of IFN-γ remain a focus area of study in literature. In this review, we summarize and explore the dualistic role of IFN-γ in regulation of tumor progression.

### Production of IFN-γ

The production of IFN-γ is mainly regulated by natural killer (NK) and natural killer T (NKT) cells in innate immunity while CD8+ and CD4+ T-cells are major paracrine sources of IFN-γ during adaptive immune response [14]. These cells are stimulated by interleukins produced in situ, such as IL-12 [15], IL-15, IL-18, and IL-21 [16], tumor- or pathogen- secreted antigens [17], and partially by IFN-γ itself through an established positive feedback loop [3]. In an inflamed or tumorous tissue microenvironment, secreted proinflammatory cytokines bind to their receptors on IFN-γ producing cells and induce the activation of transcription elements such as members of the signal transducer and activator of transcription (STAT) family, mainly STAT4 [18], T-box transcription factor (T-bet) [19], activator protein 1 (AP-1) [20], or Eomes [21] which further drive IFN-γ production. It seems that the specific transcription factor that initiates IFN-γ transcription depends on the induction signal and cell type. For example, IL-12, an interleukin secreted by antigen-presenting cells (APCs) such as macrophages, dendritic cells (DCs), and B cells, induces the activation of STAT4 in CD4+ T-cells [22]. IL-12 binds to its receptor to enhance the activity of kinases from the Janus (JAK) family, namely JAK2 and TYK2. This drives the phosphorylation of STAT4 and prompts transcriptional functions. Furthermore, STAT4 increases the expression of IFN-γ directly or indirectly, through the activation of T-bet [23]*.* In addition, Liaskou et al. reported that a low dose of IL-12 enabled STAT4 phosphorylation in regulatory CD8+ T-cells, which stimulated IFN-γ production in patients with primary biliary cholangitis [22]. On the contrary, it has been shown that cell-surface receptors such as the T cell receptor on T-cells or the NK cell-activating receptor on NK cells, recognize existing antigens and activate tyrosine kinases of the Src family. Subsequently, Src kinases stimulate mitogen-activated protein kinases (MAPKs), mostly extracellular signal-regulated kinases (ERK) and p38, which further induce Fos and Jun expression. Additionally, these transcription factors upregulate IFN-γ expression and stimulate its synthesis [24]. The secreted IFN-γ binds to its receptor (IFNGR) present on a variety of cells to regulate the immune response. Notably, IFN-γ may also stimulate APCs to secrete more IL-12 which triggers the re-activation of the IFN-γ production cycle. This is known as the positive feedback loop of IFN-γ synthesis and is detected in both tumor and inflamed environments [25].

Naïve CD4+ T-cells differentiate into helper T-cells, Th1 and Th2, in response to certain cytokines secreted during inflammation [26]. In such an environment, CD4+ Th1 cells are the main source of IFN-γ and are defined by the secretion of signature cytokines, namely IL-12, IL-2, and IFN-γ, as well as T-bet expression [27, 28]. T-bet, a transcription factor of the T-box family encoded by the *TBX21* gene, is an important promoter of IFN-γ synthesis. Its expression was initially observed in Th1 cell clones after stimulation with the anti-CD3 antibody; the same effect was absent in Th2 cells. The expression level of T-bet was correlated with the IFN-γ production in Th1 and NK cells but not in Th2 clones. In addition, the retroviral transduction of T-bet to Th2 differentiated cells could reprogram them into Th1 cells, as observed by initiation of IFN-γ production, further confirming the connection between T-bet and cytokine secretion [29].

In summary, IFN-γ is produced in response to numerous stimulants from tissue-specific environments. However, a deeper understanding of initiating signals and transcription-drivers is still needed.

### The IFN-γ signaling pathways

#### JAK-STAT pathway

As previously mentioned, IFN-γ activates its receptor composed of two subunits, IFNGR1 and IFNGR2. These subunits are intracellularly associated with kinases from the JAK family, JAK1 and JAK2, respectively. The initial interaction between IFN-γ and IFNGR activates JAKs, subsequently leading to the phosphorylation, activation, and dimerization of STAT1 transcription factors. Newly formed STAT1 homodimers then translocate to the nucleus where they anneal to the DNA sequence called IFN-γ-activated site (GAS), and initiate the transcription of numerous genes [20, 30, 31]. Full transcriptional capacity of STAT1 homodimers is achieved after interaction with co-activator proteins such as p300, cAMP responsive-element-binding protein (CBP), and minichromosomal maintenance deficient 5 (MCM5) in the nucleus [32]. The IFN-γ induced genes are named Interferon Signature Genes (ISG) which are both positive and negative regulators of inflammatory signaling [33]. In fact, many ISGs are transcription factors that further drive the transcription of effector genes. For example, interferon regulatory factor-1 (IRF-1), the member of the IRF family, is highly expressed in IFN-γ stimulated cells. Its activation induces the expression of a variety of genes involved in biological processes such as cell cycle regulation, apoptosis, growth inhibition, and tumor suppression [34]. More importantly, IRF-1 activates the synthesis of molecules associated with major histocompatibility complex (MHC) class I, which increases the sensitivity of IFN-γ-exposed cells to cytotoxic T-cell attacks [35]. In addition, it has been reported that IFN-γ can induce the expression of some immune checkpoint ligands on both tumor and T-cells via activation of JAK-STAT-IRF-1 cascade [36, 37].

**Fig. 1:**
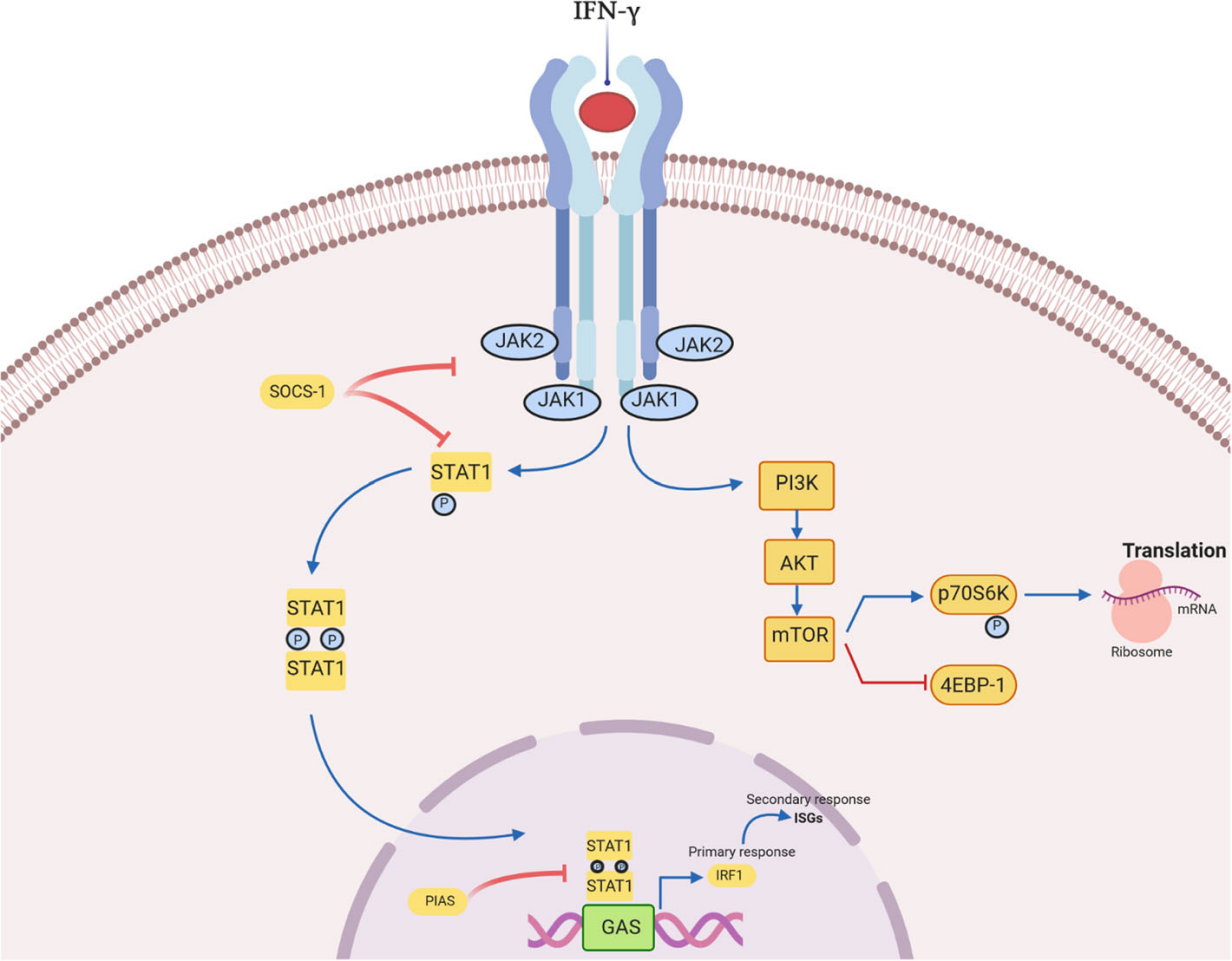
Interferon-γ signaling. Canonical IFN-γ signaling pathway requires activation of its receptor, IFN-γ receptor (IFNGR) and consequent, stimulation of JAK/STAT (Janus kinase/signal transducers and activators of transcription) signaling. The binding of IFN-γ to the IFNGR complex results in tight association of IFNGR1 and IFNGR2 and a reorientation of their intracellular domains. Close association of JAK1 and JAK2 proteins facilitate auto- and transphosphorylation and enzymatic activation. Furthermore, activated JAK proteins phosphorylate the STAT1 binding site, activating his dimerization and translocation to the nucleus where it binds to γ-activated site (GAS) elements and promotes gene transcription. The JAK-STAT pathway is negatively regulated at multiple sites: SOCS suppresses JAK and STAT activation, while PIAS inhibits IFN-γ induced gene transcription. In a non-canonical pathway, IFN-γ stimulates STAT1-PI3K-Akt axis what leads to implication of mammalian target of rapamycin (mTOR) in interferon signaling. Furthermore, mTOR/p70S6 kinase cascade promotes mRNA translation of effector proteins

The IFN-γ signaling pathway is negatively regulated by SHP phosphatases (Shp2) or proteins from the suppressor of cytokine signaling (SOCS) family, mainly SOCS1 and SOCS3 in the cytoplasm. Moreover, this pathway may be inhibited by protein inhibitor of activated STATs (PIAS) which prevents gene transcription by inducing STAT1 dephosphorylation and DNA-release [3] (Fig. 1).

Interestingly, the stimulation of widely distributed IFN-γ receptors in the human body leads to tissue specific biological effects. One of the possible explanations for this phenomenon is that IFN-γ-mediated responses are regulated by diverse signaling pathways downstream its receptor [32]. In the past few years, various signaling axes’ have been recommended as non-canonical IFN-γ stimulated, and PI3K-Akt was highlighted as the most significant signaling pathway. However, whether IFN-γ-stimulated pathways co-interact or act independently remains to be further investigated. It is important to fully understand IFN-γ signaling due to its significant implications in tumor immunity.

#### Non-canonical IFN-γ -activated pathway

Recent work from our laboratory showed that the concentration of IFN-γ in the TME of non-small cell lung cancer (NSCLC) determines which signaling pathway will be activated after IFN-γ binding to IFNGR. Song et al. reported that high doses of IFN-γ stimulated the classic JAK/STAT pathway, while low doses of IFN-γ induced the activation of ICAM1-PI3K-Akt-Notch1 signaling in cancer cells, subsequently leading to increased expression of CD133 and cancer stemness [38]. Similarly, Gao et al. explained that IFN-γ can induce the expression of PD-L1 in adenocarcinoma cell lines by activating both JAK/STAT and PI3K-Akt signaling. Mechanistically, IFN-γ binds to its receptor and then activates JAK2 to phosphorylate STAT1, and thus induces gene transcription. However, full capacity of STAT1 was PI3K-Akt dependent. The IFN-γ treated cells expressed high levels of activated and phosphorylated Akt, while the addition of a PI3K inhibitor (LY294002) resulted in a significant reduction in ISG expression, namely chemokines, CXCL9, CXCL10, and PD-L1. Therefore, the authors proposed the existence of crosstalk between these two signaling cascades in cancer cells in response to IFN-γ [39]. Another research group clarified that IFN-γ can induce the expression of carcinoembryonic antigen-related cell adhesion molecule 1 (CEACAM1) isoforms, mainly CEACAM1-L and CEACAM1-S, through the activation of PI3K-Akt-mammalian target of rapamycin (mTOR) signaling in lung epithelial cells. CEACAM1 may further promote the activity of this pathway via positive feedback loop or alternatively induce transcription and translation of inflammatory cytokines such as IL-6 and IL-8 [40]. The implication of mTOR, a serine/threonine kinase that plays an important role in promoting mRNA translation and protein synthesis [41], in IFN signaling was first described by Lekmine et al.. They explained that type I interferons (IFN-α and IFN-β) induce the phosphorylation and activation of major effector protein of mTOR, p70S6 kinase, through PI3K/mTOR cooperation while stimulating the inactivation of eukaryotic translation-initiation factor 4E-binding protein 1 (4E-BP-1), repressor of mRNA. In addition, they confirmed that IFN-γ can generate the same effects in the U2OS human osteosarcoma cell line [42, 43]. However, whether IFN-γ regulates PI3K-Akt-mTOR pathway independently of STAT signaling needs further explanation*.* In 2008, Kaur et al. reported that the disruption of Akt in mouse embryonic fibroblasts (MEF) did not influence the transcription of ISGs but led to defective mRNA translation of IFN-γ-inducible proteins. In other words, the Akt pathway augmented STAT1-induced transcription of ISGs by activating the mTOR/p70S6 kinase cascade and stimulating mRNA translation of effector proteins [44] (Fig. 1). Therefore, IFN-γ-mediated activation of PI3K-Akt axis may contribute to inflammation, translation of effector proteins and IFN-γ-dependent biological effects. However, further research is needed to fully explain the importance of detected pathway in IFN-γ signaling.

### Crosstalk between IFN-γ and immune cells

**Fig. 2:**
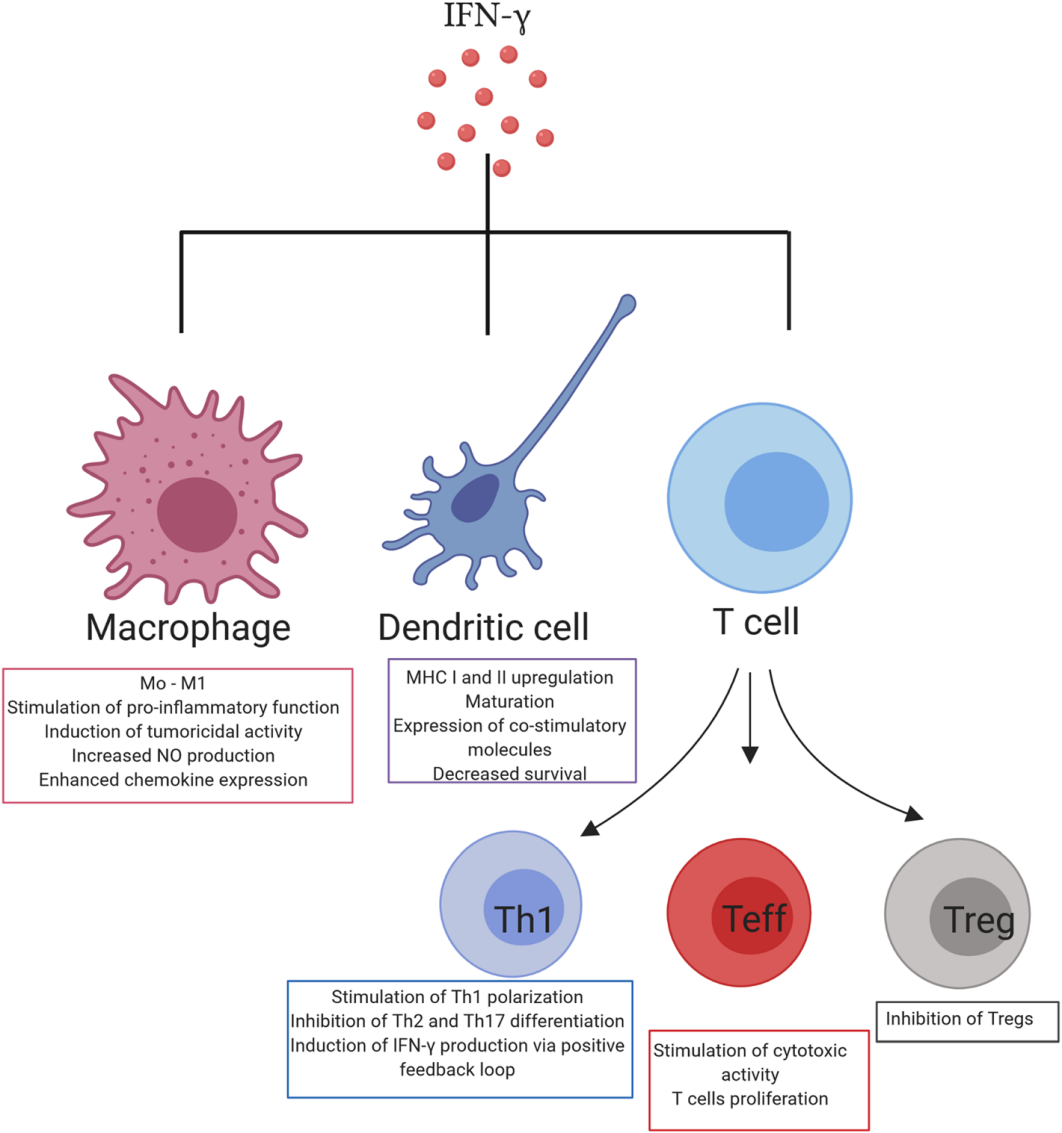
Interferon-γ interplay with immune cells. Interferon-γ interacts with IFN-γ-producing cells, such as T-cells, macrophages and dendritic cells in an inflamed or tumor microenvironment. *Macrophages*: IFN-γ stimulates polarization of macrophages toward M1 proinflammatory phenotype and enhances their capacity for chemokines secretion. *Dendritic cells*: Maturation, MHC I and II up-regulation through increased IRF1 expression, and decreased survival of dendritic cells is IFN-γ dependent. *T-cells*: IFN-γ interacts with T-cells to stimulate their differentiation toward the Th1 subset. Through a positive feedback loop IFN-γ stimulates its own production in Th1 cells and inhibits Th2 and Th17 differentiation. IFN-γ is required for maturation of naïve T-cells to effector CD8+ T-cells. The IFN-γ is main cytotoxic molecule secreted by these cells. Immune-suppressive T regulatory cells are inhibited by IFN-γ

IFN-γ is a cytokine that provides protection against diseases by acting directly on target cells or through activation of the host immune system. IFN-γ can educate immune cells to recognize and destroy pathogens; thus, understanding these interactions with host immunity is of particular importance. Besides its autocrine effects on the main IFN-γ-producing cells, IFN-γ can also act on stromal cells in an inflamed or tumor environment, such as macrophages, myeloid-derived suppressor cells (MDSC), DCs, and B cells [45] (Fig. 2). Unsurprisingly, the effect of this cytokine is mediated through the induction of numerous ISGs that define the function of immune cells. Below, we briefly summarize some of the recognized interactions between IFN-γ and immunity when exposed to pathogens or cancer.

#### IFN-γ and macrophages

It has been long appreciated that IFN-γ contributes to the innate immune response by reprogramming macrophages to the M1 proinflammatory phenotype. It is involved in “priming” macrophages by increasing their responsiveness to inflammatory molecules, such as Toll-like receptor ligands and tumor necrosis factor (TNF) [46]. In fact, Muller et al. showed that IFN-γ worked synergistically with Toll-like receptor ligands, to induce tumoricidal activity of pretreated macrophages as well as enhance nitric oxide (NO) production and the expression of proinflammatory molecules, TNFα, and IL-12 family cytokines [47]. Increased phagocytic ability, microbial and tumor cells killing ability of macrophages were achieved by IFN-γ control of specific gene expression programs involving genes related to cytokine and chemokine receptors, cell activation markers, cellular adhesion proteins, MHC proteins, proteasome formation, protein turnover, and signaling mediators and regulators [48]. Additionally, in the TME, IFN-γ produced by cytotoxic immune cells increased the number of iNOS^+^CD206^−^M1-macrophages that led to reduced tumor growth [49]. Mechanistically, iNOS induced endothelial activation by upregulating vascular cell adhesion protein-1 (VCAM-1) expression and stimulated T-cell recruitment to the tumor tissue through enhanced synthesis of the major Th1 recruiting chemokine, RANTES. Simultaneously, iNOS was shown to suppress the production of immunosuppressive and tumor growth factors [50]. As expected, iNOS^+^CD206^−^M1-macrophages were associated with favorable prognosis in a variety of tumors, such as, breast, lung, ovarian, and gastric cancer [51–54]. Therefore, IFN-γ is an important factor in the phenotypic reprogramming of macrophages into the anti-tumorigenic subset that is able to eliminate tumors.

#### IFN-γ and APCs

APCs, such as DCs and macrophages, play a role in the activation of acquired immune response by priming naïve T-cells to extracellular pathogens and tumors [3]. Interestingly, IFN-γ signaling was shown to be involved in this process by upregulating the expression of MHC I complex on both immune and non-immune cells, therefore facilitating the recognition of pathogen-derived antigens by effector T-cells (Teffs) [55, 56]. More specifically, type II interferon increases IRF1 expression, which enhances the expression of MHC class I molecules by binding to the promoter region [57]. Furthermore, IFN-γ signaling in DCs leads to their maturation, high expression of costimulatory molecules, such as CD40, CD54, CD80, CD86, and CCR7, secretion of IL-12 family cytokines together with IL-1β, and activation of both CD4+ and CD8+ T-cells [58, 59]. On the contrary, IFN-γ plays a key role in limiting antigen presentation during the chronic stage of infection by reducing the survival of DCs in a dose-dependent manner [60]. Taken together, IFN-γ not only initiates immune response by stimulating the activation of T-cells but also prevents their over-activation and exhaustion.

The ability of IFN-γ to upregulate MHC class II molecules has also been shown. Expression of the main transcription factor of MHC-II antigen presentation-class II transactivator (CIITA) is augmented in response to IFN-γ [3]. Weidinger et al. showed that in the settings of viral infection, neutralization of IFN-γ led to significant turnover of surface MHC class II, inhibition of antigen-presentation, and subsequently switch of CD4+ T-cells from immunoprotective Th1 to immunosuppressive Th2 phenotype [61]. Similarly to the induction of MHC I molecules, IFN-γ signaling in APCs led to protein kinase C mediated activation of IRF1 transcription factor that supports CIITA transcription [62]. Altogether, the crosstalk between type II interferon and APCs is necessary to initiate adaptive immune response, activate Teffs, and facilitate elimination of pathogens or antigen-producing tumor cells.

#### IFN-γ and T cells

Cross-regulation of IFN-γ and CD4+ Th1 cells is well documented. IFN-γ production is controlled by IL-12-activated Th1 cells, while stabilization of their phenotype is maintained through the release of IFN-γ [46]. Through interaction with its receptor, IFN-γ stimulates downstream signaling route, and increases STAT1 activity, which subsequently induces T-bet expression. This transcription factor suppresses Th2 and Th17 differentiation of CD4+ T-cells by inhibiting GATA3 [63]. Alternative studies suggest that T-bet redirects GATA3 transcription factor to Th1-specific binding sites, rather than block its activity. Therefore, T-bet acts at distal elements to activate and maintain its own expression even when IFN-γ is limited [19]. Moreover, T-bet drives transcription of Th1-related molecules, particularly IL-12 receptor and IFN-γ [64]. Therefore, the cyclical interplay between type II interferon and immunostimulating CD4+ T-cells has an important role in modulating inflammation. However, IFN-γ can also induce apoptosis in CD4+ T-cells, thereby impairing the CD4:CD8 ratio, and reducing secondary antitumor immune response [65].

Other subclasses of CD4+ T-cells, in particular Th2 cells, are defined by IL-4/IL-5/IL-13 production and GATA3 expression. The link between their differentiation and IFN-γ is again T-bet. Djuretic et al. reported that co-expression of T-bet and Runx3 is required for IL-4 silencing and thus control of the Th1-Th2 switch [66]. Similarly, a negative correlation was detected between the availability of IFN-γ and IL-17 producing T-cells or Th1 and Th17 subsets in inflammatory conditions [67]. One of the proposed mechanisms behind this observation lies in the ability of IFN-γ to modulate the production of IL-23, a cytokine required for optimal Th17 polarization. In mice infected with *Bacille Calmette-Guerin* (BCG), IFN-γ increased the level of IL-12 in both DCs and T-cells, and subsequently expanded the production of IFN-γ. Consequently, this limited IL-23 secretion and the frequency of IL-17-producing CD4 T-cells [68]. Another explanation is that IFN-γ inhibits STAT3 or Smad, which were shown to be essential for Th17 differentiation [69].

Naïve T-cells differentiate into cytotoxic, effector subsets when APCs present them recognizable antigens. A characteristic feature of Teff is the secretion of IFN-γ, together with cytotoxic molecules, perforin, and granzymes. Factors regulating transcription of these molecules are T-bet [70] and its paralogue-eomesodermin (Eomes) [21]. As previously explained, antigen presentation and T-bet expression are closely related to IFN-γ secretion, thus it is not surprising that this cytokine also affects Teff proliferation and function. For example, in the setting of viral infection, type II interferon stimulates the proliferation of activated CD8+ T-cells through direct interaction with its receptor on their surface [71]. Moreover, it increases their cytotoxicity via upregulation of granzyme B and TNF-related apoptosis-inducing ligand (TRAIL), which are key proteins involved in the process of apoptosis [72]. However, there are also evidences that IFN-γ can negatively impact the proliferation of Teffs [73] or limit their responses [74].

Regulatory T-cells (Tregs) are an immune-suppressive subset of T-cells whose production is essential for preventing over-activation of Teffs and tissue damage. Interestingly and in accordance with its proinflammatory role, IFN-γ was proposed as an antagonistic factor of Treg proliferation and function. For example, B cell produced – IFN-γ was shown to induce antigen-specific T- and B- cell responses while suppressing the differentiation of Tregs in arthritic mice [75]. In addition, Th1 cell differentiation can block the generation of Tregs in specific environments [76, 77]. On the other hand, Tregs limit IFN-γ production by NK cells and Teffs [78] and therefore establish a loop to accelerate their suppressive functions and dampen immune response. In summary, IFN-γ acts as an intermediate factor of complex relationships between distinct immune cells, making it particularly important in the maintenance of immune homeostasis.

### Roles of IFN-γ in cancer immunology

Recognized physiological roles of IFN-γ inspired the research community to attempt clinical application of this powerful cytokine for a variety of diseases, including cancer. However, results of tumor-related clinical trials were inconsistent and have raised some pertinent questions. ‘Does IFN-γ contribute to immune-mediated tumor regression or does it stimulate cancer growth?’ ‘Can we predict the effects of IFN-γ after introducing it to tumors?’ ‘What components of the TME interplay with IFN-γ and how do they do so?’ These and many other questions remain to be answered, if we want to understand and benefit from IFN-γ-mediated antitumor immunity. Here, we give some insights into how IFN-γ regulates cancer immunology by discussing the results of previously published studies.

### The IFN-γ-mediated antitumor effects

#### IFN-γ induces apoptosis of cancer cells

**Fig. 3:**
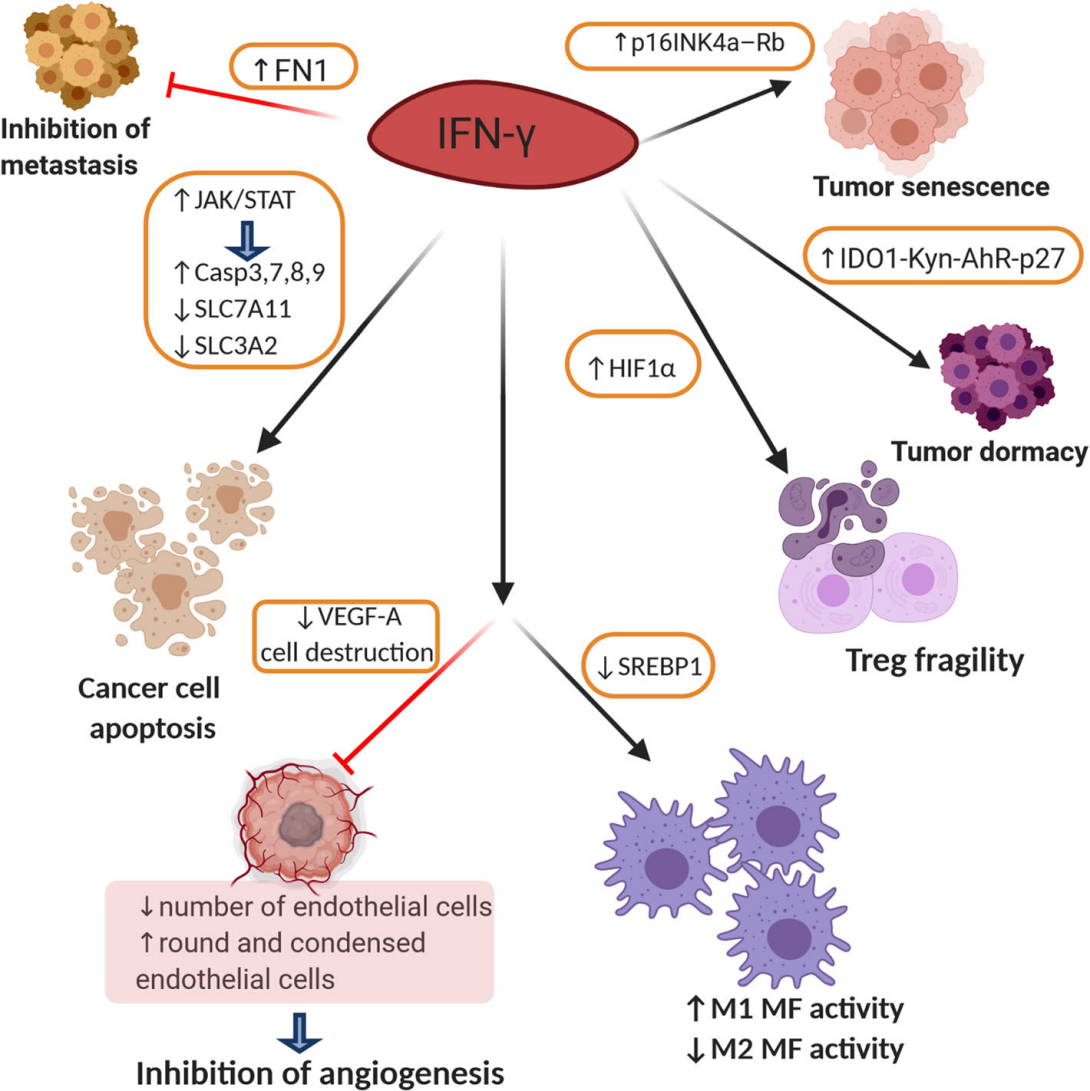
Mechanisms of interferon-γ mediated cancer regression. The IFN-γ stimulates activation of JAK/STAT signaling in the TME what further drives increased caspase activity and downregulation of SLC7A11 and SLC3A2. Consequently, cancer cells undergone apoptosis directly or through induced lipid peroxidation and ferroptosis. IFN-γ induces fragility of Tregs and inhibits the formation of tumor blood vessels by downregulating VEGF-A. Additionally, tumor angiogenesis is inhibited by IFN-γ-induced endothelial cells destruction. Through stimulation of M1 macrophage polarization and inhibition of their M2 phenotype, it contributes to effective antitumor immune response. The IFN-γ can stimulate p16INK4a-Rb pathway and thus, tumor senescence, while IFN-γ-mediated activation of IDO1-Kyn-AhR-p27 pathway shifts tumors to a dormant state. Moreover, IFN-γ increases FN1 expression that limits cancer metastasis. SLC7A11-solute carrier family 7 member 11; SLC3A2-solute carrier family 3 member 2); IDO1-indoleamine 2,3-dioxygenase 1; Kyn-kynurenine; AhR-aryl hydrocarbon receptor; p27-cyclin-dependent kinase inhibitor 1B; p16INK4a-p16(INK4a) cyclin-dependent kinase; Rb-retinoblastoma; FN1-fibronectin-1

The first promising sign that IFN-γ can be used as an antitumor agent was the discovery of its pro-apoptotic effects on cancer cells (Fig. 3). Our recent work clearly demonstrated that high doses of IFN-γ could induce apoptosis in NSCLC cell-lines, namely A549 and H460, by activating JAK-STAT1-caspase signaling. Western blot analyses showed that STAT1 forced transcription and synthesis of caspase 3 and caspase 7, which further initiated apoptotic processes in cancer cells [38]. Additionally, it was shown that IFN-γ can increase the motility of antigen-specific CD8+ T-cells to the antigen-expressing (target) cells and enhance the killing capacity of target cells. When IFN-γ^+/+^ and IFN-γ^−/−^ CD8 T-cells were incubated with the target cells authors observed significantly higher effectiveness of IFN-γ competent cells. Addition of anti-IFN-γ-antibody to the co-culture system markedly reduced target cell killing [79]. Interestingly, IFN-γ can selectively induce apoptosis in stem-like colon cancer cells through JAK-STAT1-IRF1 signaling in a dose-dependent manner. Specific sensitization to IFN-γ treatment is the consequence of higher expression of IFNGR on stem cell surface in comparison to other colon cancer cells [80]. Kundu et al. reported that precise neutralization of cytokine from IL-12 family, namely p40 monomer, induces IL-12-IFN-γ signaling cascade in prostate cancer both in vitro and in vivo, which subsequently leads to cancer cells death and tumor regression. They found that anti-p40 antibody treatment significantly elevated the expression of apoptosis-related genes such as caspase 3, caspase 7, caspase 8, caspase 9, BAD, BID, cytochrome C, BAK, and p53 [81]. Consistently, in NSCLC cells lines, namely H1975, HCC827, and H1437, IFN-γ induced programmed cell death through the activation of caspases downstream of JAK-STAT1 signaling [82]. Similar results have been reported in melanoma cells wherein the activation of caspase 3 was IFN-γ/IRF3/ISG54 dependent [83]. However, the first clinical trials in which recombinant IFN-γ was used for cancer treatment did not show promising results, only a moderate number of patients benefited, while many others experienced severe side effects [84]. IFN-γ signaling in tumor cells directly activates apoptotic processes, but non-specificity of IFNG/IFNGR interaction increases the chance for side effects. Therefore, exploring exclusivity of the IFN-γ/tumor cells/apoptosis relationship aid in the discovery of new therapeutic targets for cancer treatment.

#### Other IFN-γ-dependent tumor-suppressive mechanisms

Although, IFN-γ can directly affect the viability of tumor cells, increasing evidence points to interactions with surrounding stromal cells for effective rejection of solid tumors (Fig. 3). For instance, immunohistology analyses of large tumor sections revealed that IFN-γ could reduce the number of endothelial cells and induce blood vessel destruction and later promote tumor tissue necrosis [85]. In fact, Kammertoens et al. showed responsiveness of cancer endothelial cells by highlighting the necessary role that IFN-γ plays in the regression of solid tumors. By using electron microscopy they observed that IFN-γ-exposed endothelial cells became round, condensed, and more occluded, which reduced blood flow in tumor tissues and subsequently, prompted tumor ischemia [86]. Similarly, by interacting with stromal fibroblasts IFN-γ downregulated the expression of vascular endothelial growth factor A, a growth factor critical for tumor neovascularization [87]. Therefore, it is equally important to investigate IFN-γ-mediated effects on tumor stromal cells, especially in solid, well-established tumors.

Interplay between IFN-γ and macrophages in an inflamed setting has previously been described [88–90] and has raised questions regarding their interaction in the TME. Unsurprisingly, crosstalk between IFN-γ and M1-like immunostimulatory tumor-associated macrophages (TAMs) was sufficient to inhibit tumor growth in Lewis lung carcinoma and colon adenocarcinoma. Generated M1-like TAMs secreted CXCL9, CXCL10, and CD86, which stimulated the recruitment of cytotoxic T lymphocytes (CTLs) to the TME as well as their activation. Recruited CTLs produced IFN-γ that was proven to be critical for sustaining M1 TAM activity and tumor inhibition [91]. Reprograming of IL33^−^/^−^ Tregs was also linked to higher IFN-γ production and thus, improved the immune response in tumor tissue. Specifically, epigenetic reprogramming was detected through increased chromatin accessibility of the *IFNG* locus and elevated IFN-γ production in a nuclear factor (NF)-κB-T-bet-dependent manner [92]. In addition, it was recently reported that IFN-γ could indirectly inhibit M2-like immunosuppressive TAMs via blocking the synthesis of fatty acids. The role of IFN-γ was to downregulate the expression of sterol regulatory element-binding protein 1 [93], a transcription factor that regulates genes involved in the process of lipogenesis and glycolysis [94], which impaired TAMs function [93] (Fig. 3).

IFN-γ interacts with distinct cytokines from the TME to induce cancer growth arrest. Synergistically with TNF, IFN-γ stimulates the senescence of tumor cell growth through stabilization of p16INK4a – Rb pathway. This effect is mediated by activation of STAT1 and TNF receptor 1 and is maintained permanently in vitro and in vivo [95]. Together with inducing apoptosis or senescence, IFN-γ can shift tumors to a dormant state [96]. As recently shown IFN-γ – mediated upregulation of IDO1 increased the intracellular concentration of kynurenine (kyn, IDO1 – catalyzed tryptophan metabolite), which activated aryl hydrocarbon receptor (AhR). AhR moved to the nucleus and directly upregulated transcription of cell cycle-regulatory molecule, p27. Thus, IDO1-Kyn-AhR-p27 pathway was proposed as a mechanism which explains how high concentration of IFN-γ induces tumor dormancy [97]. The existence of IL-12-IFN-γ relationship has also been described. As the IL-12 producers, DCs stimulate NK cells to secrete IFN-γ, therefore, survival of tumor-bearing mice was improved and number of metastasis was reduced [98]. Moreover, IFN-γ produced by NK cells altered tumor structure and limited the number of metastasis by increasing the expression of the extracellular matrix protein, fibronectin 1 [99]. Their subset, invariant NK cells was also found to be an important source of IFN-γ in the TME. However, its production was limited due to lactic acid-induced inhibition of PPARγ and PLZF, which consequently diminish cholesterol synthesis, crucial for IFN-γ production. Therefore, when intratumoral invariant NK cells were treated with PPARγ agonist, authors observed amplified antitumor efficacy through the promotion of IFN-γ signaling [100] (Fig. 3).

#### IFN-γ contributes to the efficiency of cancer immunotherapy

**Fig. 4:**
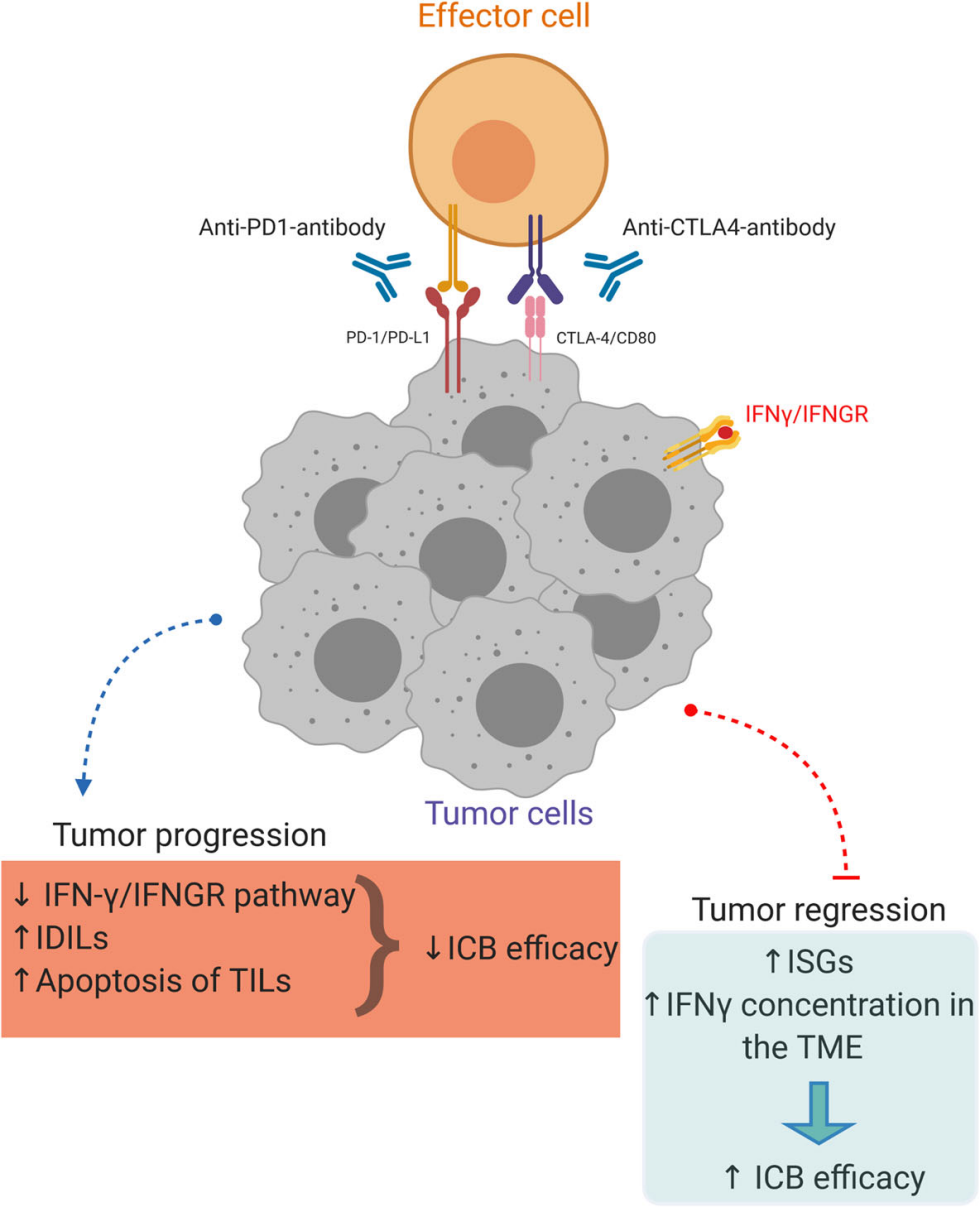
Interferon-γ contributes to cancer immunotherapy efficacy. The efficacy of anti-PD-1-antibody and anti-CTLA4-antibody in cancer treatment may be improved or diminished by the presence of IFN-γ. *Tumor progression*: The downregulation of IFN-γ/IFNGR pathway may decrease the efficacy of cancer immunotherapy. The IFN-γ drive expression of inhibitory ligands (IDILs) on tumor cells such as TNFRSF14, LGALS9, MHCII, CD86, IFIT1 and MX1 to dampen the immune response. Moreover, IFN-γ exerts apoptotic effects on immune effector tumor-infiltrating cells. *Tumor regression*: Increased expression of interferon signature genes (IFNG, CD274, LAG3, and CXCL9) or IFN-γ concentration in the TME is linked to better ICB efficacy. IFNGR-interferon gamma receptor; IDILs-interferon-driven inhibitory ligands; TILs-tumor infiltrating lymphocytes; ISGs-interferon signature genes; ICB-immune checkpoint blockade; TME-tumor microenvironment

The revolutionary discovery of antibodies targeting immune checkpoint molecules, such as programmed cell death protein 1 (PD-1), its ligand PD-L1, and cytotoxic T-lymphocyte-associated protein 4 (CTLA-4), provided hope for patients with chemo-resistant and late-stage tumors. However, their efficiency has only been proven in a small portion of treated patients [101, 102]. IFN-γ is believed to be one of the critical factors determining the success of immunotherapy (Fig. 4). By analyzing gene expression profiles from tumor tissue samples, Ayers et al. reported that metastatic melanoma, head and neck squamous cell carcinoma, and gastric cancer patients who responded to anti-PD-1 therapy had higher expression scores for IFN-γ-related genes when compared to non-responders. They proposed that the detected IFN-γ signature (IDO1, CXCL10, CXCL9, HLA-DRA, STAT1, and IFNG) can be a prediction marker for the clinical response to immune checkpoint inhibitors [103]. Similarly, a four-gene IFN-γ signature (IFNG, CD274, LAG3, and CXCL9) has been suggested as identifying pattern for urothelial and NSCLC patients who can benefit from the anti-PD-L1 antibody durvalumab [104, 105]. Moreover, successful anti-PD-1 treatment depends on intratumoral crosstalk between IL-12 and IFN-γ. After binding to PD-1, this antibody stimulates CD8+ T-cells to secrete IFN-γ, which activates its receptor on DCs, thus increasing the production of IL-12 in the TME. The newly generated interleukin acts back on CD8+ T cells to further stimulate IFN-γ production and enhance cytotoxic tumor cell function. Therefore, activation of the proposed positive feedback loop improved tumor control in mice after the administration of PD-1 antibodies [25]. Alternative mechanism by which IFN-γ contributes to efficiency of cancer immunotherapy was described by Wang et al. In that model, tumor-infiltrating CD8+ T-cells secreted IFN-γ in response to nivolumab, an anti-PD-L1 antibody. The released IFN-γ mediated lipid peroxidation and ferroptosis in tumor cells by reducing the uptake of cystine and excretion of glutamate, resulting in tumor cells death both in vitro and in vivo. Mechanistically, type II interferon activated the JAK1-STAT1 signaling pathway, which further downregulated the transcription of SLC7A11 and SLC3A2 proteins of the glutamate-cystine antiporter system (Fig. 3). Likewise, the clinical benefits of cancer immunotherapy were reduced in nivolumab-treated mice bearing INFGR^−/−^ tumors [106].

Thibaut et al. recently suggested a model in which tumor-reactive T-cells secrete IFN-γ, which diffuses extensively to alter the TME in distant areas. The prolonged activity of IFN-γ has been shown to be crucial for antitumor immune response [107] as shown by induction of PD-L1 expression and inhibition of tumor growth [108]. Furthermore, Zhang et al. proposed that IFN-γ may be a good therapeutic option for improving the efficacy of PD-1 blockade therapy for pancreatic cancer by preventing the trafficking of CXCR2+ CD68+ immunosuppressive macrophages to the TME by blocking the CXCL8-CXCR2 axis [109].

As expected, the efficiency of anti-CTLA-4 therapy was also IFN-γ dependent. Whole exome sequencing data showed that melanoma tumors resistant to immunotherapy had defects in IFN-γ signaling, namely loss of IFNGR1, IRF-1, JAK2 and IFNGR2 genes, as well as amplification of SOCS1 and PIAS4 inhibitory genes [31]. Therefore, we can propose that the combination of immune checkpoint inhibitors and IFN-γ can potentially be a good strategy to increase the overall efficiency of cancer immunotherapy. Indeed, two such clinical trials have already been initiated testing the combination of nivolumab or pembrolizumab with IFN-γ (NCT02614456 and NCT03063632, respectively). Other studies suggest that disruption of IFN-γ signaling in tumor cells could boost tumor growth and impact the efficiency of given immune checkpoint inhibitor therapy. Amplification of IFN-γ-pathway inhibitory molecules or downregulation and loss of its receptor and downstream signaling mediators are common mechanisms for tumor cells to avoid generated immune response [11]. It was recently shown that aging can also consistently attenuate IFN-γ signaling in triple-negative breast cancer patients and limit the efficiency of immune checkpoint blockade (ICB) therapy [110]. Another hypothesis is that enhanced intratumoral production of IFN-γ can improve the potency of ICB therapy in patients with cancer. For example, pharmacological blockade or partial genetic deletion of CBM complex (CARMA1–BCL10–MALT1) in Tregs re-program them to secrete IFN-γ which results in tumor regression. In addition, combination of CBM inhibition and anti-PD-1 antibodies enabled tumor control in MC38 colon carcinoma-bearing mice who were resistant to anti-PD-1 monotherapy [111]. Similarly, tumor regression has been observed only in melanoma-bearing mice treated with PD-1 targeted therapy together with antibodies against neuropilin-1. Neuropilin-1 is a protein found on most of the tumor-infiltrating Tregs, important for their suppressive function. Notably, Neuropilin-1 deletion in Tregs led to increased expression of Th1 cell markers such as T-bet and IFN-γ. Treg-secreted IFN-γ drove intratumoral fragility of the remaining immune-suppressive Tregs via hypoxia-inducible factor 1-alpha (HIF1α) which stimulated host immunity to eliminate cancer cells [112]. Similarly, it was suggested that metastatic potential of tumor cells after receiving immunotherapy was due to reduction of IFN-γ in the TME and augmented activity of integrin αvβ3 signaling axis [113]. Collectively, we can conclude that the presence of IFN-γ in the TME is required for optimal antitumor responses in cancer patients receiving mono- or combined immune checkpoint inhibitors [114–117]. IFN-γ concentration, induction of IFN-γ signature genes, and tumor/immune cell responsiveness could serve as biomarkers to predict patient response to immunotherapy; it could also highlight the need for external manipulation of IFN-γ pathway in tumor tissue [118, 119].

Collectively, we can conclude that IFN-γ contributes to tumor eradication directly or indirectly by cooperating with other members of the TME. The use of cytokine as an effective antitumor molecule could be possible if we understand how IFN-γ operates in tumor tissue. However, considering the increasing number of studies showing the tumor promoting functions of type II interferon, this will be challenging.

### IFN-γ-mediated pro-tumorigenic effects

#### IFN-γ contributes to tumor metastasis

**Fig. 5:**
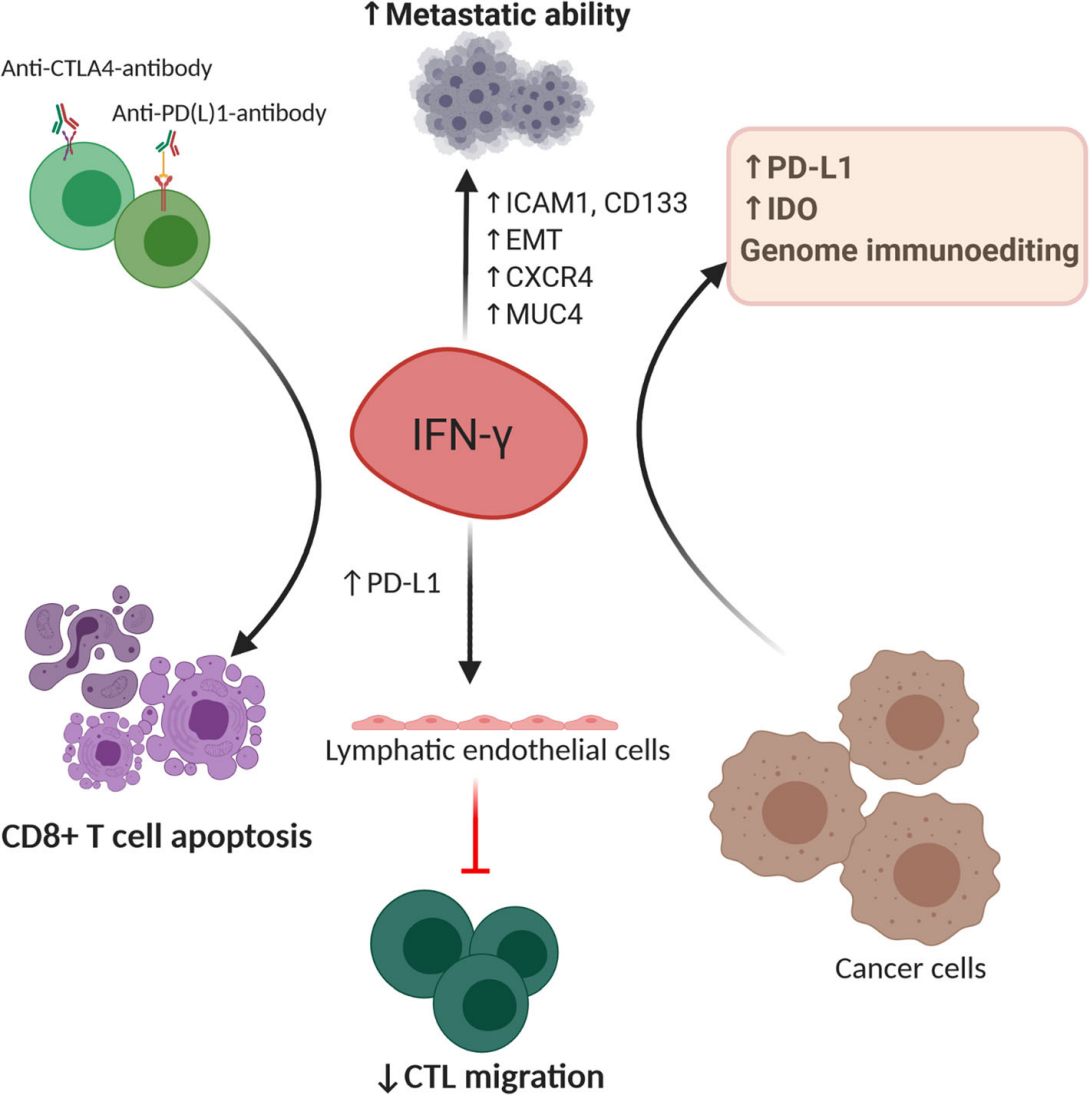
Mechanisms of interferon-γ mediated cancer promotion. Tumor metastasis may be facilitated by IFN-γ which induces the expression of ICAM1 and CD13, EMT or CXCR4 and the production of MUC4**.** Combination of anti-CTLA-4-antibody and anti-PD(L)-1-antibody may highly stimulate secretion of IFN-γ in the TME, which in turn results in CD8+ T-cell apoptosis. The IFN-γ increases synthesis of inhibitory molecules, IDO, and PD-L1, and induces genome immunoediting. High IFN-γ-stimulated PD-L1 expression on lymphatic endothelial cells prevent CTL’s migration to the TME and thus, damps immune response. EMT-epithelial–mesenchymal transition; ICAM1-intercellular adhesion molecule 1; CXCR4-C-X-C chemokine receptor type 4; MUC4-mucin 4; CTL-cytotoxic T lymphocytes

It was reported that low doses of IFN-γ generated at the site of the tumor by host-infiltrating cells or during cytokine therapy could enhance the survival of tumor cells in the circulation and enhance their metastatic potential [120]. Our recent work showed that the concentration of IFN-γ in the TME determines whether the function of the given cytokine will be pro- or anti-tumorigenic. We explained that tumors treated with low-dose IFN-γ acquired metastatic properties, while infusion with high dose led to tumor regression. When cancer cells were pretreated with low-dose IFN-γ and injected into the lateral tail vein of mice, we observed significantly larger lung metastatic nodes in comparison to cancer cells pretreated with phosphate buffer saline. The effect was ICAM1- and CD133-dependent [38]. In addition, IFN-γ contributes to the formation of a metastatic niche by transforming cancer stem cells to metastatic cancer stem cells through the induction of the chemokine receptor, namely CXCR4, which enhances their migratory and invasive potential [121]. Another prometastatic role of IFN-γ was observed in prostate cancer cells, where it promoted epithelial-to-mesenchymal transition via the activation of JAK/STAT1 signaling and induction of IFN-induced protein with tetratricopeptide repeats 5 (IFIT5). Furthermore, IFIT5 mediated the degradation of tumor-suppressive microRNA and upregulation of EMT transcription factors [122]. Similarly, significant upregulation of IFN-γ signaling correlated with IFIT5 expression in metastatic renal cell carcinoma [123]. The MUC4 mucin, a membrane-bound glycoprotein, was shown to be highly expressed in pancreatic tumors and was linked to an aggressive and metastatic tumor phenotype [124]. Interestingly, IFN-γ can stimulate MUC4 transcription by the activation of STAT1 in pancreatic cells [125], as well as in female reproductive carcinoma cell lines and endometrial cancer cells [126]. A recent study by Sing et al. explained the metastatic role of IFN-γ in triple-negative breast cancer. They revealed that the loss of the tumor suppressive transcription factor Elf5, together with its ubiquitin ligase FBXW7, could activate intrinsic IFN-γ signaling and promote tumor progression and metastasis, all through the stabilization of IFNGR1 at the protein level. Moreover, this signaling enhanced the expression of PD-L1 and led to immune suppression [127]. In contrast to previously mentioned IFN-γ-endothelial cell interactions with anti-tumorigenic consequences, evidence shows that IFN-γ-responsive pericytes accelerate the metastasis of lung carcinoma cells [128]. Therefore, it is suggested that selective activation of IFNGR on distinct blood vessel cells determines the role of IFN-γ in tumor progression (Fig. 5).

#### IFN-γ leads to immune escape

##### IFN-γ impairs T cell-immune response

Interestingly, tumor-associated lymphatic vessels also respond to IFN-γ. Accumulation of antigen-specific T-cells increase IFN-γ concentration in tumor tissue, which induce expression of PD-L1 on lymphatic endothelial cells. Consequently, this limits the migration of cytotoxic T-cell from the peritumoral space to the TME tumor microenvironment and dampens antitumor immunity [129]. Moreover, IFN-γ can induce apoptosis in tumor-specific T-cells to compromise antitumor immunity. As Pai et al. explained in a low tumor burden state, combination of anti-CTLA-4 and anti-PD-1 therapy antibodies activates T-cells to secrete high-levels of intratumoral IFN-γ, which in turn killed T cells and led to tumor immune escape [130] (Fig. 5).

##### IFN-γ induces PD-L1 and IDO expression in tumors

One of the best described pro-tumorigenic roles of IFN-γ is the induction of immune checkpoint receptor, PD-L1 and IDO in tumor tissue (Fig. 5). This may directly abrogate T-cell activity in a variety of tumors, for example, NSCLC and ovarian cancer [36, 131–135]. Kandel-Kfir et al. suggested a mechanism by which IFN-γ treated hepatocellular carcinoma (HCC) cells showed increased expression and acetylation of myocyte enhancer factor 2D which further promoted PD-L1 synthesis [136]. Observably, human tumor-specific CTLs were shown to be unable to produce the active form of IFN-γ, lowering or inhibiting CTLs response in tumor tissue. As seen, in an ex vivo model of CTL lines from cancer patients, CpG hypermethylation of the IFN-γ promoter region was inversely correlated with transcription, translation, and cytotoxicity [137]. Chronic exposure to low IFN-γ levels in H22 hepatoma, MA782/5S mammary adenocarcinoma and B16 melanoma led to tumor development and induction of PD-L1, PD-L2, CTLA-4 and Foxp3 molecules which at least partially mediated tumor immune evasion [138]. Additionally, prolonged IFN-γ signaling promoted both PD-L1 dependent and independent resistance to ICB treatment. Benci et al. observed that persistent IFN-γ signaling increased STAT1 expression in cancer cells which stimulated transcription of interferon-driven inhibitory ligands (IDILs), namely PD-L1, TNFRSF14, LGALS9, MHCII, CD86, IFIT1 and MX1. Multiple inhibition of recognized IDILs significantly improved ICB response and survival of tumor-bearing mice [139–142]. Furthermore, they proposed that the ratio of ISGs expression in immune versus cancer cells was a better prediction marker for ICB response. As explained, IFN-γ induces the expression of resistant genes in cancer cells while this signaling drives maturation of NK and innate lymphatic cells as well as synthesis of CXCL9 and CXCL10 in immune cells to enhance T-cell infiltration. Therefore, by blocking the IFN-γ pathway in tumor cells we can promote innate immune functions and improve ICB response in previously resistant tumors [143].

IDO is an enzyme involved in suppression of NK [144] and T-cell responses and in promotion of tumor immune tolerance [145]. In recent years IDO has emerged as a potent target for cancer treatment due to an increasing number of positive antitumor pre-clinical and clinical results of developed IDO inhibitors [128, 146, 147]. IDO’s relationship with IFN-γ was firstly described when Werner et al. explained that macrophages stimulated with IFN-γ were able to degrade tryptophan [148], a major IDO target [145]. Furthermore, clarifying that IFN-γ can directly induce IDO expression in tumor cells, such as lung cancer cells [149], prostate cancer cells [150], and acute myeloid leukemia blast cells [151]. Mechanistically, IFN-γ activated canonical JAK/STAT1 signaling pathway, upregulated IRF1 expression, and subsequently IDO transcription [152]. Moreover, IFN-γ mediated the differentiation of Teffs to Tregs and stimulated co-expression of IDO and PD-L1 in the TME of melanoma patients. This was shown to be associated with tumor progression due to enhanced activation of distinct immunosuppressive mechanisms [153]. Other authors have suggested that IFN-γ-stimulated DCs contribute to the induction of T-cells with regulatory activity through IDO expression [154]. Overall, we can conclude that type II interferon impacts on tumor growth not only directly but also indirectly by modulating NK and T-cell immune responses in the TME.

##### IFN-γ stimulates cancer cell immunoediting

It was proposed that IFN-γ can regulate tumor immune resistance mechanisms and thus contribute to tumor progression. In an interesting study, authors complied several mouse tumor models and found that in the presence of IFN-γ producing CTLs, cancer cells developed genetic instability. In other words, IFN-γ was critical for tumor immunoediting which supports its genetic evolution and immune escape [155] (Fig. 5).

### Conclusion and future perspectives

The IFN-γ signaling has a controversial role in regulating immune status and antitumor immunity. IFN-γ could activate IFN-γ-producing cells, such as T-cells, macrophages, and DCs, in an inflamed or tumorous microenvironment, wherein high *IFNG* expression and consequential induction of ISGs were considered as good prognostic markers and predictors of clinical response to immunotherapy. A recently published paper revealed that increasing IFN-γ dose in the TME and shifting activity toward specific cells, could stimulate host immune response and improve the efficiency of various cancer therapies, including ICBs*.* However, IFN-γ may reduce immune response and stimulate tumor progression and metastasis. Similar to other cytokines, IFN-γ induces feedback inhibitory mechanisms to suppress over-activation of the immune system, which is the link to immune escape in the TME. Therefore, we can speculate that addition of IFN-γ in the treatment of cancer patients with active immunity could be both beneficial and harmful. For example, high stimulation of IFN-γ may lead to CD8+ T-cells apoptosis or induction of PD-L1 expression on lymphatic endothelial cells to prevent CTL migration to the TME, thereby dampening immune response. Moreover, IFN-γ-driven immunosuppressive inhibitory ligands such as PD-L1 and IDO, and genome immunoediting mediated cancer immune escape. Conversely, low-dose IFN-γ produced at the tumor site induces tumor stemness and increases the risk of tumor metastasis during immunotherapy. Therefore, engagement of IFNGR on distinct tumor stromal cells, induction of ISGs, immune status of the TME, and IFN-γ concentration are recognized as critical determinants for IFN-γ-mediated outcomes. Notably, an appropriate antitumor concentration of IFN-γ has yet to be determined. It is necessary to decipher IFN-γ-dependent anti- and pro-tumorigenic effects and fully understand its role in cancer patients to reap maximum benefits for patients concerning IFN-γ-based immunotherapy. Hence, in the future, we will dedicate our work toward addressing these issues and describing the role of IFN-γ in tumor progression and/or regression.

## Data Availability

Data sharing is not applicable to this article as no datasets were generated or analysed during the current study.

## Abbreviations

AhR: aryl hydrocarbon receptor
APCs: antigen-presenting cells
CTLs: cytotoxic T lymphocytes
DCs: dendritic cells
ERK: extracellular signal-regulated kinases
ICAM1: intercellular adhesion molecule 1
ICBs: immune checkpoint inhibitors
IDILs: interferon derived inhibitory ligands
IDO: indoleamine-2,3-dioxygenase
IFNGR: interferon-γ gamma receptor
IFN-γ: interferon-γ gamma
IRF: interferon regulated factor
ISG: interferon signature genes
JAK: Janus kinase family
MAPKs: mitogen-activated protein kinases
MDSC: myeloid–derived suppressor cells
MEF: mouse embryonic fibroblasts
MHC: major histocompatibility complex
mTOR: mammalian target of rapamycin
NK: natural killer cells
NKT: natural killer T-cells
NSCLC: Non-small cell lung cancer
PI3K: phosphoinositide 3-kinases
SOCS: suppressor of cytokine signaling family
STAT: signal transducer and activator of transcription
TAMs: tumor-associated macrophages
Teffs: effector T-cells
TME: tumor microenvironment
Tregs: regulatory T-cells
TNF: tumor necrosis factor
